# Genetic parameters of birth weight trait in dromedary camels (*Camelus dromedarius*)

**DOI:** 10.1007/s11250-020-02256-z

**Published:** 2020-03-10

**Authors:** Szabolcs Bene, Ferenc Szabó, J. Péter Polgár, Judit Juhász, Péter Nagy

**Affiliations:** 1 Georgikon Faculty, University of Pannonia, Deák Ferenc str. 16, Keszthely, H-8360 Hungary; 2 Faculty of Agricultural and Food Sciences, Széchenyi István University, Vár 2, Mosonmagyaróvár, H-9200 Hungary; 3 Emirates Industry for Camel Milk & Products, Dubai-Al Ain Road, Exit 26 Umm Nahad, Dubai, United Arab Emirates

**Keywords:** Dromedary camel, Birth weight, BLUP, Heritability, Breeding value

## Abstract

Birth weight data of dromedary calves from the database of one of the world’s largest dairy herds (Dubai, UAE) were analyzed for the period from 2007 to 2018. The assessment included the data of 4124 camel calves that were classified into six ecotypes (Emirate, Emirate crossed, Black, Pakistanian, Saudi-Sudanian, and Saudi crossed). The aim of the study was to describe the heritability of birth weight of calves and the breeding value of sires. Genetic parameters of birth weight were estimated by ANOVA model and two BLUP animal models as well. The mean value of the camel calves’ birth weight was 34.75 ± 5.67 kg. The direct heritability of birth weight (*h*^2^_d_ = 0.09 ± 0.04–0.11 ± 0.03) was rather low, so was the maternal heritability (*h*^2^_m_ = 0.23 ± 0.10–0.50 ± 0.06). The maternal effect from environmental origin (*c*^2^ = 0.23 ± 0.08) far exceeded the results previously calculated in cattle. There was no difference in reliability between BLUP1 and BLUP2 models, and both of them were more accurate than the ANOVA model. Based on the results of this study, we conclude that the birth weight of dromedary calves was more influenced by the dam’s intrauterine rearing capacity and by the environment, management, and feeding of the pregnant female camels than the hereditary growth potential. Considerable differences were found among male dromedaries in their breeding values for the birth weight trait.

## Introduction

The dromedary camel (*Camelus dromedarius*) belongs, within the class of mammals (Mammalia), to the biungulate (Artiodactyla) order, the “calloused foot” animals (Tylopoda) suborder, and to the camel (Camelidae) family. The species was domesticated approximately 3000–4000 years ago on the East Coast of the Arabian Peninsula (Almathen et al. 2016). It is bred for milk, meat, and fur and used also as a pack animal (Abdallah and Faye 2012). As dromedary camels did not play an important role in intensive production until recently, information related to their production potential and performance in the scientific literature is scarce.

The birth weight of dromedary calves was the subject of numerous earlier studies that was reviewed by Tibary and Anouassi (1997). More recently, Bissa (2002) also summarized the available literature but focused mainly on ecotypes (breeds) from India. According to his results, the birth weight of dromedary calves belonging to the Bikaneri ecotype was 26–51 kg. There was also a difference between the genders, as the average of male and female calves were 38.2 kg and 37.2 kg, respectively (Bhargava et al. 1965). Similar data were published by Khanna et al. (1982) and Tandon et al. (1988), but some sources such as Ram et al. (1977) and Barhat et al. (1979) reported higher birth weight data from that region.

In contrast, dromedary calves of African ecotypes (breeds) were born with lighter weight. The birth weight of calves born in Sudan was 26.2 kg (Bulliet 1975; Babiker 1984). According to Burgemeister et al. (1975), the lowest birth weight (25.8 kg) was found in Tunisian camel calves. Dromedaries in the Middle East seem to have higher (37.4 kg) birth weight (Al Mutairi 2000) compared with the African ecotypes.

Some significant effect of various genetic (i.e., genotype, paternal, maternal) and environmental (i.e., birth year, month, weight of dam, etc.) factors on the birth weight of camel calves has been described earlier (Bhargava et al. 1965; Ram et al. 1977; Tandon et al. 1988). In contrast, Bissa et al. (2000) could not observe effect of genotype on birth weight. Also, the effect of gender was reported to be nonsignificant by Bhargava et al. (1965) and Barhat et al. (1979).

Sahani et al. (1998) reported the effect of breed as the Bikaneri ecotype (38.2 kg) had higher birth weight compared with the Jaisalmer (36.4 kg) and Kachchhi (35.1 kg) ecotypes. These results were confirmed by Bissa et al. (2000). Recently Nagy and Juhász (2019) studied the complex relationship among numerous variation factors that influence the birth weight of dromedaries. They have demonstrated that the female camel, i.e., the maternal effect, had the strongest relative influence (30.3%) on the variation in calf birth weight in this species.

There are only limited and decades old data in the literature on the heritability estimates of birth weight of dromedary calves. These data are exclusively from India and show a big variation in the heritability value among studies. Ram et al. (1977) published an *h*^2^ value of 0.02, while Barhat and Chowdhary (1980), Tandon et al. (1988), and Khanna et al. (1990) reported much higher heritability (*h*^2^ = app. 0.60). In addition, we found no reference on the breeding value estimation of male dromedaries in the relevant literature.

In other species, especially in cattle (Bene et al. 2013), much more information is available in the literature on variation factors influencing birth weight such as breed, gender, age of the dam, month of birth, etc. and on the heritability of this trait compared to the dromedary camel. Birth weight of different cattle breeds varies between 23 and 47 kg (Nugent et al. 1991; Arthur et al. 1997; Bennett and Gregory 2001; Nagy et al. 2007; Olson et al. 2009). Some authors found that the direct heritability (*h*^2^_d_) of birth weight of cattle calves was high (0.4–0.5), while the maternal heritability (*h*^2^_m_) was quite low (0.1–0.2; Legault and Touchberry 1962; Eriksson et al. 2004; Phocas and Laloë 2004). In the case of the horse, the heritability value (*h*^2^) of the birth weight was ranging from 0.2 to 0.4 (Kownacki et al. 1971; Hintz et al. 1978).

The objectives of this study were (1) to determine some genetic parameters especially heritability of birth weight of dromedary camel calves and (2) to estimate the breeding value of dromedary bulls for this trait (3) using and testing different ANOVA and BLUP models on the world’s largest available dromedary camel dataset. Based on our previous results (Nagy and Juhász 2019), we hypothesized that the birth weight of dromedary camel calves was primarily influenced by environmental factors; thus, the heritability of this trait may be low.

## Material and methods

### Location of the study, animals, and general farm management

The study was conducted over 11 breeding seasons from 2007 through 2018 at the premises of Emirates Industry for Camel Milk and Products (EICMP), the world’s first large-scale camel dairy farm that is located in Dubai, United Arab Emirates (N25°, E55°). During this period, a total of 58 male (bull) and 2087 female (dam) dromedaries were included into the breeding program and 4124 progeny (calves) delivered on the farm (Table 1).

**Table 1:** Composition of the examined population

Designation	Number of animals
Number of live calves born with records	4124
Sires	58
Dams	2087
Paternal grand sires	7
Maternal grand sires	22
Total grand sires	29
Paternal grand dams	9
Maternal grand dams	269
Total grand dams	278
Calves without performance	0

Female dromedaries were kept in groups of 5 to 50 camels in open paddocks throughout the year. Bulls were housed in individual open paddocks. Daily feed consisted of alfalfa and Rhodes hay (*Chloris gayana*) with or without wheat bran and formulated feed in different quantities depending on age, production, reproductive status, and body condition of the animals. Water and mineral licking stones were available ad libitum. The breeding season extended from September until June with a peak from December to January. Further details on farm management have been described previously (Nagy et al. 2013).

Camels were categorized into 6 well-distinguishable ecotypes (Emirati, Emirati-cross, Black, Pakistani, Saudi/Sudanese, and Saudi-cross) based on geographical origin, color, appearance, and body conformation (Abdallah and Faye 2012; Fábri 2018). At the time of parturition, the camels (dams) were between 3 to 24 years of age (mean ± SE, 10.8 ± 0.1 years) and had variable parities (1 to 6).

### Collection of reproductive data, estimation of genetic parameters, and breeding value of males

For each parturition, all relevant information was recorded such as female camel and bull number, date of last mating/conception, type of breeding (natural mating or embryo transfer), date and time of birth, type of delivery, gender and weight of the calf, status of the calf (alive or dead), and time to sitting position and to standing of the calf. In this work, we only considered gestations with normal parturition and viable newborns.

Birth weight was considered as character of the calf. To estimate the genetic parameters of birth weight trait, three different models (Szőke and Komlósi 2000) were designed: one was ANOVA (Type III) model and two BLUP (*best linear unbiased prediction*; Henderson 1975) animal models. The details of the three models are summarized in Table 2.

**Table 2:** Models used for the estimation of genetic parameters for the birth weight trait in dromedary camels

Used models	Classes	ANOVA	BLUP1	BLUP2
Random effects				
- Sire (male camel)	58	+	+	+
- Animal (newborn calf)	4124	–	+	+
- Dam (cow, female camel)	2087	–	+	+
Fixed effects				
- Ecotype of dam	6	+	+	+
- Parity of dam	5	+	+	+
- Breeding season	11	+	+	+
- Month of mating	9	+	+	+
- Sex of calf	2	+	+	+
Other effects				
- Maternal genetic effect	2087	–	+	+
- Maternal permanent environmental effect	2087	–	–	+

+/–, the model includes/does not include this effect

ANOVA model did not contain information on the dam; BLUP1 model contained total maternal effect (genetic and environmental maternal effects together); BLUP2 model contained genetic and environmental maternal effects separately. It was supposed that BLUP1 model would support the previous results of Nagy and Juhász (2019) on maternal effects, while BLUP2 model could prove the magnitude of the presumed environmental effects. The models were constructed as follows:

$$ \mathrm{ANOVA}:{\hat{y}}_{\mathrm{i}\mathrm{jklmn}}=\mu +{S}_{\mathrm{i}}+{E}_{\mathrm{j}}+{P}_{\mathrm{k}}+{Y}_{\mathrm{l}}+{M}_{\mathrm{m}}+{I}_{\mathrm{n}}+{e}_{\mathrm{i}\mathrm{jklmn}} $$

where *ŷ*_ijklmn_ = birth weight of calf from “i” sire, in “j” ecotype, in “k” parity, in “l” season, in “m” month, and in “n” sex; *μ* = overall mean value; *S*_i_ = random effect of sire; *E*_j_ = fix effect of ecotype; *P*_k_ = fix effect of parity of dam; *Y*_l_ = fix effect of season; *M*_m_ = fix effect of month of mating; and *I*_n_ = fix effect of sex of calf; *e*_ijklmn_ = residual.

$$ \mathrm{BLUP}1:\hat{y}={X}_{\mathrm{b}}+{Z}_{\mathrm{u}}+{W}_{\mathrm{m}}+\mathrm{e} $$

$$ \mathrm{BLUP}2:\hat{y}={X}_{\mathrm{b}}+{Z}_{\mathrm{u}}+{W}_{\mathrm{m}}+{S}_{\mathrm{pe}}+e $$

where *ŷ* = vector of observation – birth weight of calf); *b* = vector of fixed effects (ecotype, parity of dam, season, month of mating, and sex of calf); *u* = vector of random effect (animal); *m* = vector of maternal genetic effect; *pe* = vector of maternal permanent environmental effect; *e* = vector of random residual effect; *X* = matrix of fixed effects; *Z* = matrix of random effects; *W* = matrix of maternal genetic effect; and *S* = matrix of maternal permanent environmental effect.

To determine the most suitable model for estimating the parameters, the *e*^2^ values and log-likelihood values (− 2 log L) for the three different models were compared (Bouwman et al. 2010; Alves et al. 2018).

The examined fix (environmental) factors were as follows: ecotype of dam (6 classes, as above), parities of dam (5 classes, 1–5), breeding season (11 classes), month of mating (9 classes, excluding June, July, and August), and the sex of the calf (2 classes, male or female) (Gregory et al. 1995; Van Vleck et al. 1996; Lee et al. 1997). BLUP models contained pedigree information for sire, dam, grandparents, maternal genetic effect, and maternal permanent environmental effect as random effects. Covariant was not included into the models.

Breeding value (BV) of the dromedary sires for birth weight trait was estimated with all three models (ANOVA, BLUP1, BLUP2). BV was considered as a double of the realized progeny difference (RPD), namely, BV = 2 RPD. The RPD was defined as the difference of the mean value of the birth weight data of close relatives (progeny, sibs, and halbsibs) of a particular dromedary sire and the mean value of the birth weight data of the contemporary calf group. Breeding values were estimated only for sires (*n* = 18) with at least 100 progeny.

Variance, covariance, correlation, heritability, and breeding values according to the abovementioned three models were evaluated as described by Willham (1972), Trus and Wilton (1988), and Lee et al. (1997). HARVEY (Harvey 1990), DFREML (Meyer 1998) and MTDFREML (Boldman et al. 1993) softwares were used for the estimation.

Between the birth weight of calf trait and the gestation length trait, simple Pearson’s phenotypic correlation coefficient was calculated.

## Results

The weight of healthy, newborn calves was recorded for 4124 parturitions. The descriptive statistical parameters of birth weight trait are shown in Table 3. Mean (±SD) birth weight of dromedary calves was 34.75 ± 5.67 kg, and the weight was ranging from 10 to 64 kg (CV = 16.3%). The distribution of birth weight data was not normal, but the homogeneity of variance was confirmed. A significant but weak correlation (*r* = 0.14; *p* < 0.01) was found between the birth weight and gestation length of dromedary camels. This is the reason why gestation length was not considered as a covariant in the various models.

**Table 3:** Descriptive statistics of birth weight trait

Parameters	Birth weight
N	4124
Mean (kg)	34.75
Standard error (kg)	0.09
Standard deviation (kg)	5.67
Coefficient of variation (%)	16.31
Median (kg)	35.00
Range (kg)	54
Minimum (kg)	10
Maximum (kg)	64
95% Populations boundaries (kg)	20.50–49.00
Kolmogorov-Smirnov test^†^ (*p*)	0.000
Levene test § (*p*)	0.347

^†^if *p* > 0.05, the normal distribution is confirmed; ^§^if *p* > 0.05, the homogenity is confirmed

Genetic parameters estimated with the three models (ANOVA, BLUP1, and BLUP2) are summarized in Table 4. These data were used for the calculation of breeding values. According to the previous assumption, the direct heritability of the birth weight of dromedary calves was fairly poor (*h*^2^_d_ = 0.09 ± 0.04–0.11 ± 0.03) with all three models. Hence, the contribution of the direct genetic variance to the phenotypic variance was much lower than that of the environmental variance. There was difference in magnitude of the maternal heritability between the two BLUP models. When permanent maternal environmental effects were not included in the model (BLUP1), the maternal heritability value was quite high (*h*^2^_m_ = 0.50 ± 0.06). However, when this effect was included in the model (BLUP2), the maternal heritability value was much lower (*h*^2^_m_ = 0.23 ± 0.10), but still exceeded direct heritability (*h*^2^_d_) more than twice. The correlation estimated between direct and maternal genetic effects seemed to be negative and quite weak (*r*_dm_ = − 0.33 ± 0.23 and − 0.23 ± 0.31). In addition, because the SE values were too high, the *r*_dm_ values were not reliable and informative. The maternal environmental effect estimated by the BLUP2 model is fairly high (*c*^2^ = 0.23 ± 0.08) that indicates poor genetic but strong environmental (management, nutrition, season, etc.) effects of the gestation period on the birth weight of dromedary calves.

**Table 4:** The estimated genetic parameters for the birth weight trait in dromedary camels

Parameter	Birth weight		
	ANOVA	BLUP1	BLUP2
*σ*^2^_d_ additive direct genetic variance	2.39	2.67	2.58
*σ*^2^_m_ maternal genetic variance	–	12.39	5.73
*σ*_dm_ direct maternal genetic covariance	–	−1.90	−0.90
*σ*^2^_pe_ maternal permanent environmental effect	–	–	5.68
*σ*^2^_e_ residual variance	23.80	11.85	11.70
*σ*^2^_p_ phenotypic variance	26.19	25.00	24.78
*h*^2^_d_ direct heritability	0.09 ± 0.04	0.11 ± 0.03	0.10 ± 0.03
*h*^2^_m_ maternal heritability	–	0.50 ± 0.06	0.23 ± 0.10
*r*_dm_ direct maternal genetic correlation	–	−0.33 ± 0.23	−0.23 ± 0.31
*c*^2^ the ratio of the permanent environmental variance to the phenotypic variance	–	–	0.23 ± 0.08
*e*^2^ the ratio of the residual variance to the phenotypic variance	0.81 ± 0.04	0.47 ± 0.03	0.47 ± 0.03
*h*^2^_m_ + *c*^2^	–	–	0.46
*h*^2^_T_ total heritability	–	0.24	0.17

Breeding values of dromedary sires for birth weight estimated by ANOVA and BLUP models are summarized in Table 5. Apparently, there were differences between the breeding values of the same sire depending on the model used. Namely, breeding values for direct genetic effects estimated by the ANOVA model were generally lower compared with values obtained by the BLUP models. However, these differences did not influence the ranking of the sires by their breeding value. Notable differences (3.23–4.69 kg) in breeding values for birth weight can be observed only among sires that are far up and down in the ranking away from the mean value.

**Table 5:** Breeding values of the evaluated dromedary sires for birth weight

Identity number of sire^§^	Ecotype of sire	*N*	Breeding values for birth weight by methods of estimation (kg)				
			ANOVA	BLUP1		BLUP2	
				Direct effect	Maternal effect	Direct effect	Maternal effect
2016	Black	101	+ 0.95	+ 2.21	+ 1.38	+ 2.31	+ 0.89
2021	Pakistani	336	+ 1.12	+ 2.05	+ 0.18	+ 2.11	− 0.05
2043	Emirati-cross	101	+ 0.21	+ 1.32	− 0.94	+ 1.30	− 0.45
2010	Emirati	166	+ 0.06	+ 0.83	+ 3.45	+ 0.91	+ 2.35
2053	Saudi/Suadanese	107	+ 0.15	+ 0.38	+ 0.79	+ 0.43	+ 0.53
2017	Emirati-cross	174	− 0.09	+ 0.27	− 0.48	+ 0.32	− 0.26
2015	Saudi/Suadanese	101	− 0.38	+ 0.08	− 1.18	+ 0.10	− 0.75
2045	Emirati-cross	103	+ 0.06	− 0.02	+ 0.02	− 0.08	+ 0.03
2026	Emirati-cross	256	− 0.04	− 0.17	− 3.38	− 0.10	− 1.48
2040	Emirati-cross	113	− 0.25	− 0.34	+ 0.24	− 0.15	+ 0.05
2020	Saudi/Suadanese	183	− 0.78	− 1.11	+ 1.27	− 1.07	+ 0.56
2013	Emirati	146	− 1.11	− 1.37	+ 1.49	−1.35	+ 1.13
2028	Saudi-cross	200	− 1.07	− 1.31	+ 0.93	− 1.35	+ 0.47
2001	Saudi/Suadanese	206	− 1.41	− 1.45	+ 2.30	− 1.38	+ 1.66
2027	Saudi/Suadanese	162	− 0.98	− 1.71	+ 1.22	− 1.63	+ 0.57
2000	Saudi/Suadanese	265	− 1.53	− 1.96	+ 3.27	− 1.89	+ 2.87
2011	Emirati	136	− 1.78	− 2.32	− 0.83	− 2.32	− 0.41
2004	Saudi/Suadanese	237	− 2.11	− 2.43	+ 1.24	− 2.38	+ 0.98

*N* number of progeny; ^§^males sorted by direct breeding value of BLUP2 model

Concerning the breeding values for maternal genetic effects, results obtained by two BLUP models were different. Breeding value data obtained with the BLUP1 model were higher than that obtained with BLUP2. Also, there were differences between the breeding values for direct genetic and maternal genetic effects of the same sire. In general, the breeding values obtained for maternal genetic effect were higher than that obtained for direct genetic effects. Also, the range of breeding values was different between the two models. Namely, the difference between the first and last sire in the rank was 6.38 kg and 4.35 kg when estimation was done with the BLUP1 model and with the BLUP2 model, respectively.

## Discussion

Published data on the heritability of the birth weight of different ecotypes or breeds of dromedary camels are rather limited and heterogeneous. Results of our study using the world’s largest dromedary camel dataset provide new information that can help breeding programs and sire selection in this species. This research extended the results of a recent multi-trait study (Nagy and Juhász 2019) which suggested that the maternal effect on the phenotypic birth weight of dromedary camel calves was much higher than that in the case of cattle (Bene et al. 2013) or horse (Bene et al. 2014). That is why, we assumed that the maternal effect could be well determined and resolved with the help of genetic parameters. Our study confirmed this assumption as we have demonstrated that maternal heritability was higher compared to that described in other species (Eriksson et al. 2004; Phocas and Laloë 2004).

Average birth weight of camel calves in our study was in the middle of the wide range of means (from 26 to 51 kg) that had been reported in the literature (Ram et al. 1977; Tibary and Anouassi 1997; Khanna et al. 1982; Bissa 2002). Compared to other large domestic species, the birth weight of dromedary calves is similar to that of cattle calves (Arthur et al. 1997; Bennett and Gregory 2001; Olson et al. 2009), but smaller than the birth weight of foals (Hintz et al. 1979; Kavazis and Ott 2003; Ringler and Lawrence 2008). However, it is important to note that the length of gestation of dromedary camels is much longer (approximately 384 days), and therefore, the intrauterine fetal growth rate is slower compared to that in cattle and horse (Nagy and Juhász 2019).

We evaluated different models to estimate genetic parameters and breeding values of individual dromedary bulls for the birth weight trait. According to the *e*^2^ and − 2 log L data (Alves et al. 2018), the BLUP1 and BLUP2 models were equally reliable and were more accurate than the ANOVA model. In addition, the difference in maternal genetic effects obtained by two BLUP models was due to the fact that the BLUP1 model did not contain permanent environmental effects, so, the total maternal (genetic + environmental) effect was considered as a genetic influence. Therefore, as the BLUP2 model differentiates between maternal genetic and maternal environmental effects, it seems to be more appropriate for estimating genetic parameters and breeding values for birth weight in this species.

The results of this study for direct heritability values of birth weight of dromedary calves correspond to the result of Ram et al. (1977), who published quite low values. On the contrary, some authors (Tandon et al. 1988; Khanna et al. 1990) reported much higher values of direct heritability compared with our findings. It is also interesting to note that direct heritability values obtained in our study are considerably lower than those published for cattle and horse (Kownacki et al. 1971; Hintz et al. 1978; Eriksson et al. 2004; Phocas and Laloë 2004).

The relationship between direct and maternal genetic effects (*r*_dm_) shows similar tendency as it was published for beef cattle (Bene et al. 2010). However, most other studies reported stronger correlation between direct and maternal genetic effects compared with our findings (Cubas et al. 1991; Iwaisaki et al. 2005, etc.).

In this study, the maternal environmental effect (*c*^2^ = 0.23 ± 0.08) on the birth weight of dromedary calves was higher than that published for cattle. Namely, Phocas and Laloë (2004) reported 0.02–0.04 *c*^2^ values for birth weight in French beef cattle breeds. In addition, the maternal environmental effects for weaning weight were also low (*c*^2^ = 0.00–0.14) in Angus, Hereford, Simmental, and Piedmontese breeds (Núnez-Dominguez et al. 1993; Lee et al. 1997; Pariacote et al. 1998; Carnier et al. 2000).

Based on the abovementioned findings, we conclude that the birth weight of dromedary calves was more influenced by the dam’s intrauterine rearing capacity (maternal genetic effect) and by the environment, management, and feeding of pregnant female camels (maternal permanent environmental effect) than the hereditary growth potential (additive direct genetic effect) of the calf. Most likely, this is the result of the fact that until recently no proper selection and specific breeding programs were applied in this species to improve its production potential (Hermas 1998). In agreement with this assumption, data on grandparents were insufficient in our study as it is demonstrated in Table 1. Consequently, camel breeds with stabile genetic background were not developed. Despite important phenotypic variations among dromedary ecotypes/breeds (Vijh et al. 2007; Abdallah and Faye 2012), camel genotyping studies revealed low divergence at DNA level and close genetic distance between breeds in several countries (Mehta et al. 2006; Al-Swailem et al. 2007; Spencer and Woolnough 2010; Mahrous et al. 2011). These studies show that dromedary camel ecotypes are not as heterogeneous by their genotype as cattle or horse breeds despite the fact that these species had been under stringent breeding programs for centuries and are supported by well-established official records of pedigree.

To our best knowledge, breeding value estimates for birth weight or for any other trait have not been published for the dromedary camel until now. Therefore, from the population genetics point of view, the results of this study on breeding values of individual male animals can be considered as pioneer information. As a result, dromedary bulls were ranked according to their direct and maternal genetic effects that are of great significance for the development of dromedary breeding. These results also highlight the importance of maintaining genetic diversity within the dromedary camel population. However, we also have to note that breeding values might have some limitations in this species. Our data were obtained in one well-managed dromedary camel population. But, due to the interaction of genotype by environment, the ranking of these dromedary males could have been different under different management conditions. We assume that genetic evaluation of dromedary sires in one particular environment may not be an accurate predictor of progeny performance in another environment.

In this study, we provided new data on genetic parameters of birth weight trait using the world’s largest dromedary camel dataset. Heritability values for the evaluated trait advanced our understanding of the interaction between genetic and environmental effects. Breeding value data allowed the ranking of dromedary bulls and also draw the attention to the genetic diversity of the dromedary population. The reasonable genetic and, to some extent, more environmental variance and breeding value data indicate the heterogeneity and selection possibility of the dromedary camel population offering a good opportunity both for genetic and environmental improvement. Future studies should be directed towards defining similar genetic parameters and heritability estimates for many other traits in this species. The present experience using various methods for genetic analysis for birth weight, as a model trait, will be beneficial for future studies.
